# Efficient Chemo-Enzymatic Transformation of Animal Biomass Waste for Eco-Friendly Leather Production

**DOI:** 10.3390/molecules24162979

**Published:** 2019-08-16

**Authors:** Roberto Sole, Lorenzo Taddei, Clizia Franceschi, Valentina Beghetto

**Affiliations:** 1 Dipartimento di Scienze Molecolari e Nanosistemi, Università Ca’ Foscari Venezia, via Torino 155, 30170 Mestre, Italy; 2 Codyeco S.p.a., Via del Grano 8, S. Croce sull’Arno, 56029 Pisa, Italy; 3 Ilsa S.p.a, Via Quinta Strada 28, Arzignano, 36071 Vicenza, Italy

**Keywords:** enzymatic hydrolysis, biopolymers, retanning agents, leather production, circular economy, green chemistry

## Abstract

Enzymatically processed animal biomass derived from treated bovine hides (wet blue scraps) is herein used as building block for the synthesis of a novel biopolymer. An enzymatic hydrolysis process allows to produce water-soluble lower molecular weight proteins (Bio-A), which are then reacted with glycerol and maleic anhydride (MA) in order to obtain a new intermediate (Bio-IA). With Bio-IA in hand, co-polymerization in the presence of acrylic acid is then carried out. Hydrolysed biomass, intermediates and the final biopolymer (Bio-Ac) have been characterized by means of NMR, FTIR and GPC analysis. Bio-Ac shows good performance when used as retanning agent to produce leather. Physical and mechanical properties of the leather treated with Bio-Ac have been compared with acrylic resin retanned leather, showing similar performance. The reported protocol represents an environmental-friendly interesting alternative to traditional petrochemical based retanning agents, commonly used by the leather industry.

## 1. Introduction

Tanning of hides and skins is one of mankind’s oldest trades. The EU leather industry contributes considerably to the social and economic scenario, counting nearly 24,000 companies and 400,000 employees, with a turnover of more than 31 billion Euros [1,2]. Currently, the tanning of hides involves a set of sequential steps starting from salted hides. Tanning, retanning, and fat-liquoring are the most relevant transformations in the process and may require the use of polymeric materials. Today, over 85% of the hides processed worldwide are tanned with basic chromium sulphate due to its very high efficiency, low cost and good quality of the leather obtained. Nevertheless, harmless Cr(III) present in the slurries may be oxidized to toxic and harmful Cr(VI) species. Further drawbacks derive from the production of salts, mainly chlorides and sulphates, which contribute to high values of salt concentrations in the discharged wastewater [2,3,4]. On the blacklist of chemicals commonly used in the leather industry, petrochemical based reagents occupy some of the top positions. Significant quantities of synthetic tannins, resins and aldehydes, are used daily because of their low cost and high quality of the leather obtained. Rationalising these observations, a sustainable alternative to replace these chemicals is required by leather industry. 

In this frame, industry in general is nowadays transitioning towards greener production to address environmental issues, as the demand for ecological alternatives increases, prompted by European directives in favour of the circular economy [2,5,6]. Thus, raw hides and skins, by-products of slaughterhouses, must be correctly characterized in terms of environmental footprint, to grant them an allocation in accordance with their impact.

Having in mind the circular economy principles we identified the possibility to recover and reuse metal-containing tanned leather scraps to produce biopolymers to be employed as retanning and fattening agents an interesting environmental challenge. In the last decade the recovery, recycle and reuse of industrial wastes as renewable feedstocks has been one of the main challenges of green chemistry and the circular economy [7].

As far as animal-derived biomass is concerned, a high amount of collagen-containing by-products are produced yearly as post-tanning waste. Although many studies and developments for the use of leather waste have been reported, higher value polymeric products for cosmetic and fine chemical applications are normally obtained starting from untanned collagen scraps or trimmings [8,9,10]. Chrome tanned leather scraps and shavings are instead commonly converted into fertilizers by thermal or enzymatic transformation [10,11,12,13,14,15,16]. This hydrolysed collagen may also be employed for the production of low-cost surfactants, high chrome fixation auxiliaries, fillers for paper, etc. [9,10,16,17,18,19]. Few higher value applications have been developed for the use of chrome tanned hydrolytes in combination with oxazolidines or acrylates as retanning agents [20,21].

**Scheme 1:**
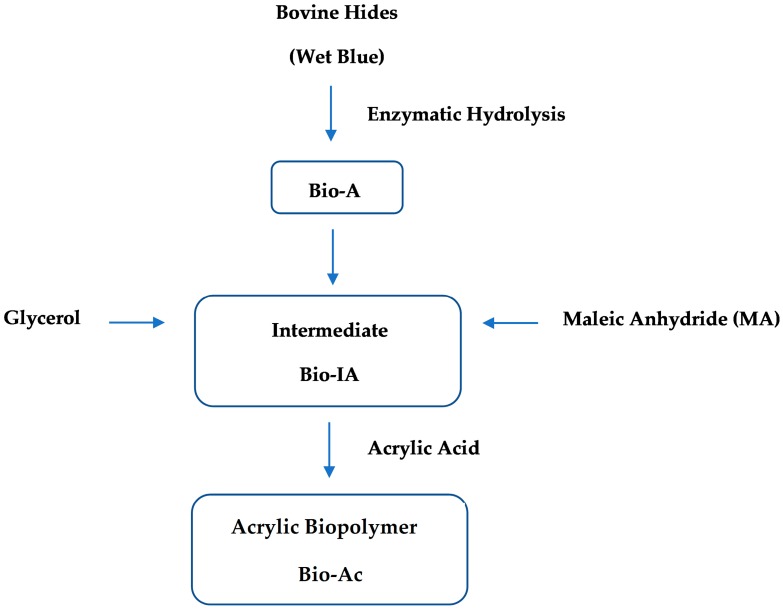
Synthetic process for the synthesis of Bio-Ac.

With all this in mind, in this work we wish to report a combined enzymatic and chemical protocol which employs tannery by-products as feedstock to produce high value biopolymers. In particular, the action of different enzymes on chrome tanned bovine leather scraps to produce hydrolysed biomass was tested. The low molecular weight water-soluble hydrolysates have then been employed as building blocks to produce high value biopolymers for retanning. To the best of our knowledge this is the first example of a combined chemo-enzymatic transformation of animal biomass waste to produce biopolymers for the leather industry. The novelty of the work here reported relies in the use of enzymatically hydrolysed chrome tanned collagen waste as a building block for the production of biopolymers for the leather industry. The overall industrial process to generate a biobased acrylic polymer (Bio-Ac) is reported in Scheme 1. According to this strategy, hydrolysed proteins derived from leather bovine scraps (Bio-A) are first reacted with glycerol and MA. Afterwards, a radical copolymerization is achieved in aqueous media in the presence of acrylic acid until complete conversion to Bio-Ac is observed.

## 2. Results and Discussion

The work here reported foresees three different steps for the processing of animal biomass which will be described separately in the sections below.

### 2.1. Enzymatic Hydrolysis

Chrome shavings recovered at the end of the tanning process were obtained and used as raw material to provide hydrolysed water soluble collagen (Bio-A). Biomass was selected according to availability of the feedstock, standardization of the biomass and analytical issues. This biomass is widely available and does not present standardization problems. For these reasons, it represents an ideal substrate to process via enzymatic hydrolysis. Fully Controlled Enzymatic Hydrolysis (FCEH^®^) is an alternative to chemical hydrolysis which can be applied to raw materials of animal or plant origin, allowing the recovery of peptides and polypeptides in good yields, while preserving the chirality of the amino acids. Enzymatic digestion is an interesting technology to recover animal hydrolysates. Since the 1980s this technique has been commercially exploited. The first example reported was applied to produce instant whipping agents for pastry [22].

In the present work, a FCEH^®^ process was conducted to produce Bio-A. Following standard literature protocols [23,24] bovine leather scraps were dispersed in water inside a stirred bioreactor equipped with temperature, weight and pH control. The selected enzymatic pool, made up of specific proteolytic enzymes, was introduced in the reactor. The mixture was kept under stirring at 50–60 °C for up to 12 h, depending on the type of enzyme and characteristics of the desired finished product. Then, the liquid suspension was subjected to centrifugation, clarification and filtration. The liquid fraction thus obtained was finally introduced in a falling film vacuum evaporation plant until the desired concentration was achieved to obtain a hydrosoluble powder. Data obtained after the hydrolysis for Bio-A are listed in Table 1. Hydrolysed biomass analysis was subjected to elemental analysis with titration to determine free amino acid content (Table 2) and microbiological tests for bacterial contaminants.

### 2.2. Characterization of Bio-A, Bio-IA and Bio-Ac

#### 2.2.1. Elemental Analysis

Enzymatic hydrolysis on Bio-A proceeded with a hydrolysis degree (HD) of 9.2%, in accordance to similar hydrolytic processes reported in literature [25]. HD indicates the intensity of the hydrolysis process and it’s calculated as the ratio between number of cleaved peptide bonds and the total number of peptide bonds. This kind of hydrolysis allows to recover almost free-chromium proteins, since Cr (III) level was detected at very low concentrations (40 mg/Kg). Harmful Cr (VI) was not detected. Proof of microbiological stability is given by the absence of *Salmonella* and coliforms.

#### 2.2.2. NMR Studies

Collagen is a protein made up of α- and β-amino acids linked by peptide bonds. Some of the amino acids present in the collagen structure, such as for example glutamic acid, arginine and lysine have more than one carboxylic or amino functional group, so unreacted pendant COOH and NH_2_ functionalities are present. ^1^H-NMR signals of the hydrolysate protein Bio-A prevalently derive from glycine moieties linked to other amino acids, mainly proline (Pro) and hydroxyproline (Hyp) [26]. As expected for complex molecules, the ^1^H-NMR spectrum of the hydrolysed protein Bio-A shows numerous unresolved signals, as reported by Vatansever et al. [27].

**Figure 1:**
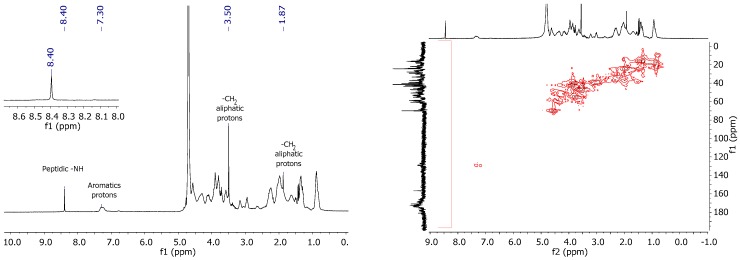
^1^H- and ^1^H-^13^C HMQC spectra of hydrolysed collagen protein in D_2_O.

The signals between 0.7 and 4.7 ppm may be attributed to different CH_2_ moieties due to the various amino acids present in the biopolymer. A relevant signal is the singlet at 8.4 ppm, which is zoomed in the upper left corner of Figure 1 and is assumed to correspond to the -NH amide protons of the protein from animal biomass. A ^1^H ^13^C HMQC NMR experiment carried out on Bio-A further highlighted that no coupling between the proton at 8.4 ppm and a carbon atom is present, so the signal may be attributed to NH amide protons. A weak unresolved multiplet between 7.1 and 7.4 ppm is characteristic of the aromatic part of amino acids present in Bio-A (phenylaniline, tyrosine).

**Scheme 2:**
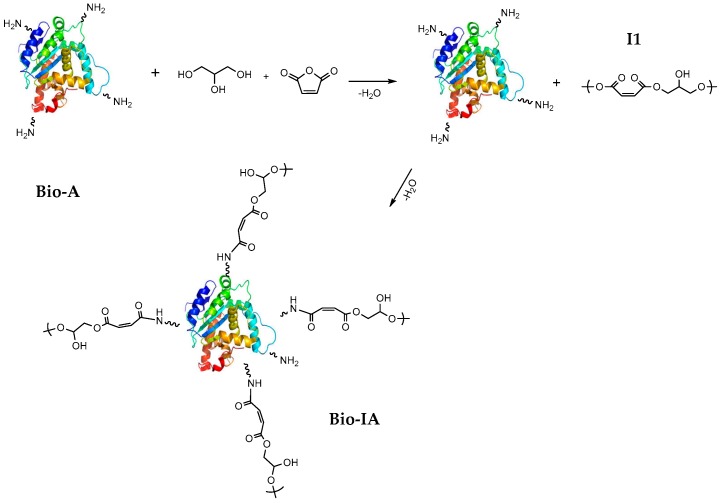
Supposed reaction mechanism between glycerol, maleic anhydride and Bio-A.

According to the synthetic strategy reported in Scheme 2, Bio-A is then reacted with glycerol and MA to give intermediate Bio-IA. Different experiments were carried out in the presence of variable amounts in *wt*% of glycerol, MA and Bio-A. The first experiments carried out with ratios of glycerol/MA between 10/90 and 45/55 *wt*/*wt*%, gave very viscous mixtures which could not be easily recovered from the reaction vessel. Thus, for all further tests a glycerol/MA ratio ≥50/50 *wt*/*wt*% was employed.

It is important to note that glycerol is known to stabilize the triple-helical structure of solubilized collagen due to the formation of hydrogen bonds and other low energy interactions modifying the solvation shell of the protein [28]. In fact, glycerol is a common plasticizer for polymers and specifically its effect is supposed to inhibit the self-association of collagen, increasing the stability of the protein; nevertheless, no reaction should occur between the protein and glycerol. To verify this hypothesis, equal amounts of Bio-A and glycerol where mixed together at room temperature and the ^1^H-NMR spectrum of the sample recovered corresponds to a mixture of the unreacted starting materials, confirming that no reaction takes place.

**Figure 2:**
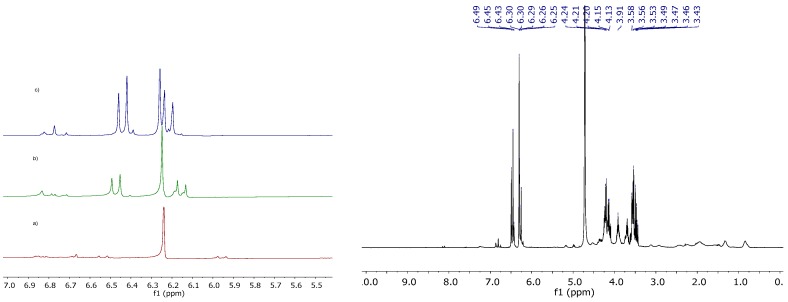
On the left are depicted part of the ^1^H-NMR spectra of Bio-IA samples prepared with (a) Bio-A/glycerol/MA = 70/15/15 *wt*%, (b) Bio-A/glycerol/MA = 50/25/25 *wt*%, (c) Bio-A/glycerol/MA = 30/35/35 *wt*% and on the right side, the full spectrum of Bio-IA.

From the comparison of the ^1^H-NMR spectra of three Bio-IA samples prepared with different *wt*% of Bio-A/glycerol/MA (see Figure 2) it appears that a set of doublet of doublets due to the olefinic protons of the maleate ester in the region between 6.00 and 6.55 ppm becomes more and more intense as the amount of glycerol and maleic anhydride increase. In accordance with the literature [29], an acid catalysed reaction between MA and glycerol proceeds to form the corresponding maleate ester I1 (Scheme 2).

These data moreover substantiate the hypothesis that the condensation between glycerol and MA proceeds faster than the reaction between MA and Bio-A. The maleate ester I1, can then react with free amine groups present in Bio-A to give a Bio-A-maleate (Bio-IA) building block (Scheme 2). ^1^H-NMR of Bio-IA is reported in Figure 2.

**Figure 3:**
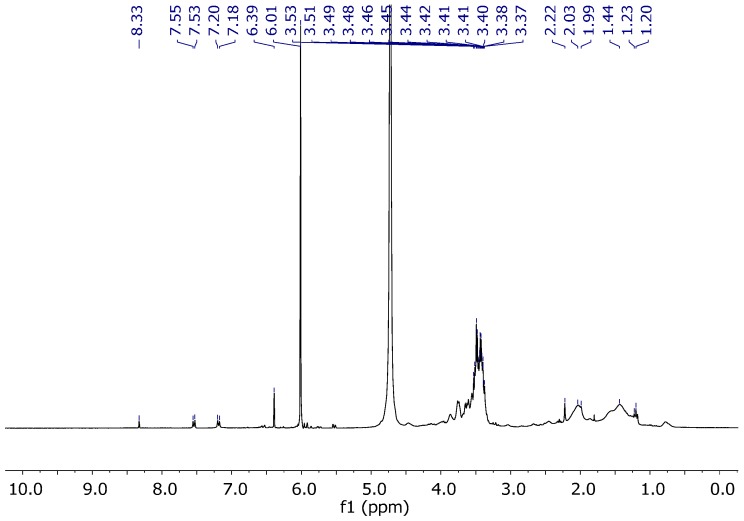
^1^H-NMR of Bio-Ac.

When a higher amount of MA was used, crystallization of unreacted maleic anhydride was observed, leading to gradual decomposition of Bio-IA. For this reason, all further tests were carried out with a ratio Bio-A/glycerol/MA = 30/35/35 *wt*%. Bio-Ac is achieved via copolymerization of Bio-IA with acrylic acid (10 *wt*%), in the presence of a radical initiator (hydrogen peroxide), according to standard procedure [30]. The ^1^H-NMR spectrum of Bio-Ac (Figure 3) shows characteristic polyacrylate signals between 1.25 and 2.5 ppm [31]. It is possible to assume that Bio-IA is fully converted to the polymerized product, since maleate olefinic signals between 6.00 and 6.55 ppm, are no longer observed.

#### 2.2.3. FTIR Analysis

**Figure 4:**
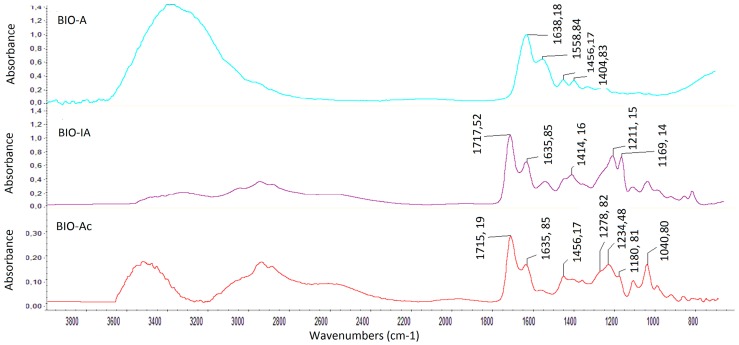
FTIR spectra in ATR mode of Bio-A, Bio-IA, Bio-Ac.

In accordance to literature [32,33,34,35] the FTIR spectrum of Bio-A (blue spectrum, Figure 4) is mainly characterized by C=O stretching (1700–1640 cm^−1^) and N-H bending (1570–1550 cm^−1^) absorbances. Bio-IA (red spectrum, Figure 4) shows a broad signal between 3100 and 2800 cm^−1^, characteristic of COOH functional groups together with C=O stretching adsorptions at 1718 cm^−1^ and 1214 cm^−1^, confirming the presence of maleate ester [36]. FTIR adsorptions of the amide groups of the protein are only slightly affected, in agreement with NMR data. In accordance to literature [37] absorptions due to C-O stretching (1200–900 cm^−1^) and C-O-C stretching (1200–1100 cm^−1^) were assigned to the glycerol moiety.

#### 2.2.4. GPC Studies

GPC analysis were performed on hydrolysed proteins (Bio-A), intermediate (Bio-IA) and the final biopolymer (Bio-Ac), as reported in Table 3. The average molecular weight (Mw) of Bio-A is 5149 Da, a value which is close to data reported in the literature for hydrolysed proteins derived from bovine hides [38]. Functionalization of Bio-A with glycerol and MA allows to increase Mw to 7722. This means that, according to the reaction mechanism supposed in Scheme 2, I1 reaches a molecular weight around 2.5 KDa. Finally, racial co-polymerization of Bio-IA leads to the formation of Bio-Ac, having a Mw of approximately 42,000 Da.

### 2.3. Characterization of Bio-Ac Retanned Leather

#### 2.3.1. Application Tests: Leather Retanning

Bio-Ac was tested on conventionally chrome tanned bovine leather as a retanning agent. The haptic and physical characteristics of the crust Bio-Ac leather were compared to crust leather achieved using conventional retanning products such as syntans and acrylic resins (Table 4). All the post-tanning steps involve the fixation of a solute in solution onto a solid (collagen hide). We may assume that the diffusion and penetration of Bio-Ac into the collagen may be described, in accordance to literature [26], as a two-step process defined as follows: (i) transfer of the reagent from the solution to the substrate (collagen hide); (ii) formation of different interactions or bonds (hydrophobic, electrostatic, covalent) between the reagent and the solid collagen. The diffusion and penetration of the retanning agent will be influenced by the chemical nature of the solute, by the pH at which the treatment is carried out and by the tanning agent employed. In a pH range between 5.0 and 7.0 Bio-Ac may be assumed to work in a similar manner to an anionic retanning agent having good filling power and grain fixation [39,40].

Preliminary physical mechanical tests, reported in Table 4 and Table 5, were selected in order to verify the quality of Bio-Ac crust for automotive application. Presently the automotive compartment is one of the most appealing markets for green and sustainable tanning systems.

Data obtained show that Bio-Ac retanned leather has very good light fastness, probably due to a low concentration of reactive functional groups on the absorbed biopolymer such as for example carbon-carbon double bonds, which are responsible for color changes over time [38]. Refractometric fogging is very similar to both acrylic and phenolic leather products, while gravimetric fogging is moderately lower. Organoleptic characteristics of Bio-Ac crust reveal a higher grain tightness and fullness but an overall lower softness. These phenomena may be explained, in accordance to literature [41], assuming that the absorption of Bio-Ac influences the thickness of the collagen fiber, reducing their mobility hence, increasing tightness and fullness while reducing softness. The dyeing is somewhat more intense compared to references.

Although mechanistic studies are beyond the scope of this work, it may be inferred that the retanning action of Bio-Ac is favoured by the acrylic moiety present in the polymer which favours the fixation of Bio-Ac to the amine functional groups available in the hides after chrome tanning [24,40]. This hypothesis seems to be in good agreement with the physical mechanical tests reported in Table 5 and with SEM images (see below), which show a similar behaviour of Bio-Ac to retanning acrylic resins more than to phenolic syntans. These results confirm that the biopolymer may be suitable for producing leather for automotive use, which requires low emission levels to ensure a healthy vehicle cabin environment [42].

#### 2.3.2. SEM Analysis

**Figure 5:**
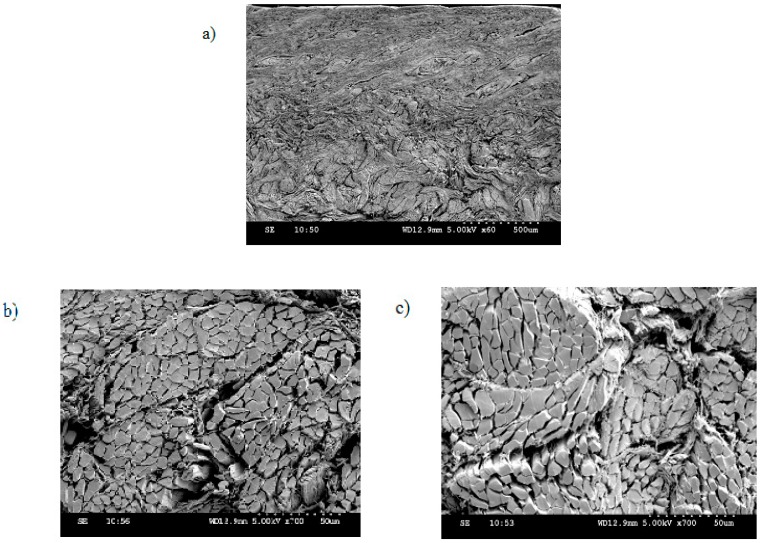
Leather sample cross sections at low magnification (**a**, ×60) and higher magnification (**b–c**, ×700).

Crust leather samples prepared by standard chrome tanning and Bio-Ac retanning, exhibit a clean grain surface, and in general, SEM observations are in good agreement with analogous data reported in literature [43,44,45,46]. At low magnification (Figure 5, picture a) it is possible to observe the grain at the top of the image. Fibre structure appears to be in good conditions, well opened without looseness visible. SEM images (Figure 5, pictures b,c) show tightly packed, smoothly oriented fibres and well aggregated collagen fibers (increasing the grain leather strength) which agree with the overall considerations reported above regarding tightness and fullness.

## 3. Materials and Methods

### 3.1. Materials

All reagents were purchased from Sigma-Aldrich (St. Louis, MO, USA) and used without any purification. Chrome tanned collagen scraps were obtained from treated bovine hides (wet blue scraps) and furnished by Codyeco S.p.A. (S. Croce sull’Arno, PI, Italy). ILSA S.p.a. (Arzignano, VI, Italy) provided hydrolysed Bio-A. Bio-IA and Bio-Ac were synthetized by Codyeco S.p.A.

### 3.2. Methods

Elemental analysis was performed for hydrolysed Bio-A proteins. Ashes were determined by weight loss at 105 °C and at 550 °C respectively (Reg.CE 152/2009 IO-CEN); organic matter (OM) by loss in ignition (OM = dry matter–ash); pH in water (3/50, *w/v*); total carbon (TC) by wet oxidation method with potassium dichromate (MI 1.2.9-Ed.2 Rev.2:2017); total nitrogen (TKN, MI 1.2.9–Ed.2 Rev.2:2017) via the Kjeldhal method; ammonium nitrogen (NH_4_^+^ -N) by extraction with diluted HCl and steam distillation with magnesium oxide (UNI EN 15475:2009); total organic nitrogen (TON) by difference (TON = TKN–NH_4_^+^ -N). Hydrolysis degree has been calculated following known method (G.U. 26/01/01 n°21, DM 21/12/00). Microbiological stability of the hydrolysed product was evaluated through salmonella (DM 1337 27/01/2014 GU 42 29/02/2014) and coliforms (ISO 4832:2006) content. Total metals were determined by acid digestion with ultrapure nitric acid and hydrogen peroxide (Merck, Darmstadt, Germany) in Teflon bombs in the microwave oven (Milestone, Sorisole, BG, Italy) and determination by Microwave Plasma Atomic Emission Spectrometry (MP_AES, Agilent, Santa Clara, CA, USA). Post-retanned leather samples were examined using a scanning electron microscope (SEM; S-3000 N, Hitachi, Chiyoda, Tokio, Japan) operating at 5 kV.

### 3.3. Enzymatic Hydrolysis

Bio-A supplied by ILSA S.p.A. was produced through an advanced fully controlled enzymatic hydrolysis process (FCEH^®^) using a combination of proteolytic enzymes (endo- and exo-proteases) derived from non-genetically modified organisms. Extraction is followed by a concentration phase and spray-drying in order to obtain a hydrosoluble powder. Stability of the product is ensured both by the origin of the byproducts and by the specific plant process.

### 3.4. Structural Characterization

FTIR spectra were recorded by using a Thermo Nicolet Protegè 460 spectrometer (Thermo Fisher Scientific, Waltham, MA, USA), in a range frequency between 4000–400 cm^−1^. All the spectra have been collected in ATR mode. Elemental analysis was performed using the official analytical protein methods according to the corresponding Italian regulation (D.Lgs. 75/2010) and European Regulation (CE 2003/2003). Gel Permeation chromatography (GPC) was performed for Bio-A, Bio-IA and Bio-Ac with a Yarra sec 2000 column (300 × 7.8 mm, Phenomenex, Bologna, BO, Italy). Calibration curves were btaned using a Gel Filtration Molecular Weight Marker Kit for Molecular Weights 6500–66,000 Da (Catalogue Number MWGF60, Sigma Aldrich). ^1^H- and HMQC ^13^C-NMR spectra were recorded on an AVANCE 300 spectrometer (Bruker, Billerica, MA, USA). The chemical shift values of the spectra are reported in δ units with reference to the residual solvent signal. The proton assignments were performed by standard chemical shift correlations.

### 3.5. Retanning Tests

General procedure for the retanning tests: soaked chrome tanned bovine leather (wet blue) has been retanned according to conventional method, employing Bio-Ac, acrylic or phenol retanning agents and natural fat liquoring agents to produce crust upper leather. These leather samples were employed for physical-mechanical tests. Light fastness of retanned leather was rated (from 1 to 5) by visual assessment of experienced tanners.

## 4. Conclusions

Enzymatically hydrolysed proteins derived from industrial waste biomass are used in this study as starting material for the synthesis of a new acrylic biopolymer (Bio-Ac). Enzymatic hydrolysis allowed to recover water soluble lower molecular weight proteins which have been firstly reacted with glycerol and maleic anhydride. Afterwards, copolymerization with acrylic acid leads to the final biopolymer. Each synthetic step has been optimised by varying reaction parameters and the products fully characterised by NMR and FTIR spectroscopy. Molecular weights were calculated by means of GPC chromatography, showing a final Mw of 42,400 Da. Application of Bio-Ac as retanning agent has been successfully implemented. SEM analysis and physical tests on leather retanned with Bio-Ac show similar quality and performances of leather treated with commonly used fossil fuel derived chemicals. Based on these results, we believe that Bio-Ac represents the first example of a biomass derived biopolymer used as retanning agent for a more sustainable tannery process in agreement to the principles of circular economy. Further different biomass sources will be evaluated in order to provide an improved protocol for the synthesis of biomass derived biopolymers as retanning agents together with LCA data.

## Figures and Tables

**Table 1:** Elemental analysis of Bio-A.

Analysed Parameter	Bio-A
Organic nitrogen (N) %	15.4 ± 0.3
Total carbon (C) %	42.6 ± 1.0
Dry matter %	94.2 ± 2.0
Ashes %	6.6 ± 0.5
pH	5.7 ± 0.5
Total aminoacids %	96.8 ± 3.0
Free aminoacids %	1.8 ± 0.4
Hydrolysis degree	9.2 ± 1.1
Cr (III) (mg/Kg)	40 ± 2
Cr (VI) (mg/Kg)	<0.5 ^(a)^
*Salmonella* spp	ABSENT
Coliforms (UFC/g)	<10

^(a)^ Quantification limit of the instrument.

**Table 2:** Analysis of the aminoacid composition of Bio-A.

Aminoacids ^(a)^	Total (%)	Free (%) ^(b)^	Aminoacids ^(a)^	Total (%)	Free (%) ^(b)^
Hydroxyproline	11.0	<lq	Cysteine	<lq	<lq
Aspartic acid	4.9	<lq	Tyrosine	1.0	<lq
Serine + asparagine	3.4	0.1	Hydroxylisine	1.2	<lq
Glutamic acid	9.2	0.2	Valine	2.3	<lq
Glycine	22.2	0.2	Metionine	0.9	<lq
Hystidine + glutamine	0.9	<lq	Ornitine	0.5	<lq
Arginine	8.2	0.1	Lysine	3.4	<lq
Threonine	1.1	1.0	Isoleucine	1.4	<lq
Alanine	7.9	0.1	Leucine	2.9	<lq
Proline	12.5	0.1	Phenilalanine	1.9	<lq
γ - Aminobutyric acid	<lq	<lq	Tryptophan	<lq	<lq
α - Aminobutyric acid	<lq	<lq	Total	96.8	1.8

^(a)^ Aminoacids were determined by electron spray technique (ESI-MAS) using analytical method IDL 1.2.23. ^(b)^ lq: quantification limit: 0.1%.

**Table 3:** MW data acquired for biomass intermediates and biopolymers.

Sample	Mw (Da)
Bio-A	5149
Bio-IA	7722
Bio-Ac	42,400

**Table 4:** Physical Tests for Crust Leather.

Recipe ^a^	Retanning	Light Fastness ^b^	Fogging Refractometric ^c^	Fogging Gravimetric ^d^
Acrylic Biopolymer	Bio-Ac (6%)	5	99	0.8
Standard	Acrylic resin (6%)	4	96	2.3
Standard	Phenolic Syntan (6%)	4	94	3.5

^a^ Wetting back: ethoxylated surfactant (0.3%); fatliquoring: sulphited oil (3%), dyeing: acid brown 425 (4%); % based on wet blue weight; ^b^ Light fastness 72 h/BST 50 °C (blue wool scale) measured according to UNI EN IOS 105-B02; ^c^ Fogging refractometric 6 h/75 °C (%) measured according to ISO 17071 A; ^d^ Fogging gravimetric 6 h/100 °C (mg) measured according to ISO 17071 B.

**Table 5:** Mechanical Tests for Crust Leather.

Recipe ^a^	Retanning	Grain Distension ^b^		Grain Strength ^b^		Tear Strength ^c^
		Elongation (nm)	Load (Kg)	Elongation (nm)	Load (Kg)	
Acrylic Biopolymer	Bio-Ac (6%)	8.36	18	12.55	40	0.8
Standard	Acrylic resin (6%)	8.73	19	11.65	30	2.3
Standard	Phenolic Syntan (6%)	6.39	16	10.67	48	3.5

^a^ Wetting back: ethoxylated surfactant (0.3%); fat-liquoring: sulphited oil (3%), dyeing: acid brown 425 (4%); % based on wet blue weight; ^b^ Grain distension and grain strength measured according to EN IOS 3379; ^c^ Horizontal and vertical tear strength measured according to EN ISO 3377-2; all values are the result of measurements on three different samples.

## References

[1] Conseil National du Cuir. https://conseilnationalducuir.org/en/press/releases/2018-01-24.

[2] Black M., Canova M., Rydin S., Scalet B.M., Roudier S., Sancho D.L. (2013). Best Available Techniques (BAT) Reference Document for the Tanning of Hides and Skins. Industrial Emissions Directive 2010/75/EU.

[3] Ilou I., Souabi S., Digua K. (2014). Quantification of Pollution Discharges from Tannery Wastewater and Pollution Reduction by Pre-Treatment Station. Int. J. Sci. Res..

[4] Beghetto V., Lodovico A., Gatto V., Samiolo R., Scrivanti A. (2019). Sustainable use of 4-(4,6-dimethoxy-1,3,5-triazin-2-yl)-4-methylmorpholinium chloride as metal free tanning agent. J. Clean. Prod..

[5] European Commission (2010). Being Wise with Waste: The EU’s Approach to Waste Management.

[6] Communication from the Commission to the European Parliament, the Council, the European Economic and Social Committee and the Committee of the Regions (2015). Closing the Loop—An EU Action Plan for the Circular Economy.

[7] Pfaltzgraff L.A., De Bruyn M., Cooper E.C., Budarin V., Clark J.H. (2013). Food waste biomass: A resource for high-value chemicals. Green Chem..

[8] Catalina M., Cot J., Balu A.M., Serrano-Ruiz J.C., Luque R. (2012). Tailor-made biopolymers from leather waste valorisation. Green Chem..

[9] Sundar V.J., Gnanamani A., Muralidharan C., Chandrababu N.K. (2011). Recovery and utilization of proteinous wastes of leather making: A review. Rev. Environ. Sci. Biotechnol..

[10] Sundar V.J., Raghavarao J., Muralidharan C., Mandal A.B. (2011). Recovery and Utilization of Chromium-Tanned Proteinous Wastes of Leather Making: A Review. Crit. Rev. Environ. Sci. Technol..

[11] Cabeza L.F., Taylor M.M., DiMaio G.L., Brown E.M., Marmer G.L., Carriò R., Celma P.J., Cot J. (1998). Processing of leather waste: Pilot scale studies on chrome shavings. Isolation of potentially valuable protein products and chromium. Waste Manag..

[12] Jiang H., Liu J., Han W. (2016). The status and developments of leather solid waste treatment: A mini-review. Waste Manag. Res..

[13] Dang X., Yang M., Zhang B., Chen H., Wang Y., Mandal A.B. (2019). Recovery and utilization of collagen protein powder extracted from chromium leather scrap waste. Environ. Sci. Pollut. Res..

[14] Pahlawan I.F., Sutyasmi S., Griyanitasari G. (2019). Hydrolysis of leather shavings waste for protein binder. Proceedings of the IOP Conference Series: Earth and Environmental Science.

[15] Narasimhan K., Sehgal P.K., Joseph K.T. (1980). Condensation of aromatic sulfonic acid with hydrolysates from leather wastes. J. Am. Leather Chem. Assoc..

[16] Zehra B., Nawaz H.R., Solangi B.A., Nadeem U., Zeeshan M. (2014). Preparation of double action surfactant using protein hydrolyzate from fleshing waste and its utilization as a lubricant with retanning property in leather making. Proc. Pak. Acad. Sci..

[17] Santos L.M., Gutterres M. (2007). Reusing of a hide waste for leather fatliquoring. J. Clean. Prod..

[18] Munoz J.M., Maldonado M.V., Rangel A.S. (2002). Development of a tanning process based on using hydrolyzated material collected from leather scrap. J. Am. Leather Chem. Assoc..

[19] Yılmaz O., Kantarli I.C., Yuksel M., Saglam M., Yanik J. (2007). Conversion of leather wastes to useful products. Resour. Conserv. Recycl..

[20] Afsar A., Aslan A., Gulumser G., Ocak B. (2010). A study on usability of collagen hydrolysate along with oxazolidine in leather processing. Text. App..

[21] Zehra B., Nawaz H.R., Solangi B.A., Nadeem U. (2019). Extraction of protein from chrome shavings, modification with acrylic monomers and further re-utilization in leather processing. Am. Sci. Res. J. Eng. Technol. Sci..

[22] Gunther R.C. (1979). Chemistry and Characteristics of Enzyme-Modified Whipping Proteins. J. Am. Oil Chem. Soc..

[23] Kanagaraj J., Velappan K.C., Chandra Babu N.K., Sadulla S. (2006). Solid wastes generation in the leather industry and its utilization for cleaner environment—A Review. J. Sci. Ind. Res..

[24] Taylor M.M., Dieffendorf E.J., Na G.C. (1990). Enzymic treatment of chrome shaving. Leather Manuf..

[25] Cavani L., Margon A., Sciubba L., Ciavatta C., Marzadori C. (2017). What we talk about when we talk about protein hydrolizate-based biostimulants. AIMS Agric. Food.

[26] Convington A.D. (2009). Tanning Chemistry: The Science of Leather.

[27] Vatansever B., Binici B. (2015). A quantitative method for the measurement of hydrolysed type-I collagen protein in dietary supplement syrup using HPLC-SEC-UV technique. J. Chem. Metrol..

[28] Penkova R., Goshev I., Gorinstein S., Nedkov P. (1999). Stabilizing effect of glycerol on collagen type I isolated from different species. Food Chem..

[29] Valerio O., Pin J.M., Misra M., Mohanty A.K. (2016). Synthesis of Glycerol-Based Biopolyesters as Toughness Enhancers for Polylactic Acid Bioplastic through Reactive Extrusion. ACS Omega.

[30] Ristić I., Miletić A., Cakić S., Govedarica O., Janković M., Sinadinović-Fišer S., Budinski-Simendić J. (2016). The synthesis of polyacrylic acid with controlled molecular weight. Phys. Chem..

[31] Molnar R.M., Bodnar M., Hartmann J.F., Borbély J. (2009). Preparation and characterization of poly(acrylic acid)-based nanoparticles. Colloid Polym. Sci..

[32] Doyle B.B. (1975). Infrared Spectroscopy of Collagen and Collagen-Like Polypeptides. Biopolymers.

[33] Vidal B., Mello M.L.S. (2011). Collagen type I amide I band infrared spectroscopy. Micron.

[34] Camacho N.P., West P., Torzilli P.A., Mendelsohn R. (2001). FTIR Microscopic Imaging of Collagen and Proteoglycan in Bovine Cartilage. Biopolymers.

[35] Riaz T., Zeeshan R., Zarif F., Ilyas K., Muhammad N., Safi S.Z., Rahim A., Rizvi S.A.A., Rehman I.U. (2018). FTIR analysis of natural and synthetic collagen. Appl. Spectrosc. Rev..

[36] Lisperguer J., Nunez C., Perez-Guerrero P. (2013). Structure and thermal properties of maleated lignin-recycled polystyrene composites. J. Chil. Chem. Soc..

[37] Salehpour S., Dubé M.A. (2012). Reaction Monitoring of Glycerol Step-Growth Polymerization Using ATR-FTIR Spectroscopy. Macromol. React. Eng..

[38] Castiello D., Chiellini E., Cinelli P., D’antone S., Puccini M., Salvadori M., Seggiani M. (2009). Polyethylene-Collagen hydrolizate thermoplastic blends: Thermal and mechanical properties. J. Appl. Polym. Sci..

[39] Zhang Y., Wang L. (2009). Recent research progress on leather fatliquoring agents. Polym. Plast. Technol..

[40] Liquiang J., Yanwei W., Yulu W., Yanchun L. (2014). Preparation and application of an amphiphilic acrylic copolymer as a retanning agent. Sltc J..

[41] Bianli R., Qi F., Yuye C., Yunjun L., Xianglong Z. (2018). The relantionship between the organoleptic properties of leather and the aggregate structure of collagen fibres. J. Soc. Leather Technol. Chem..

[42] UL LLC (2015). Vehicle Interior Air Quality: Addressing Chemical Exposure in Automobiles.

[43] Krishnamoorthy G., Sadulla S., Sehgal P.K., Manda A.B. (2013). Greener approach to leather tanning process: D-Lysine aldehyde as novel tanning agent for chrome-free tanning. J. Clean. Prod..

[44] Nashy E.H.A., Hussein A.I., Essa M.M. (2010). Retanning agents for chrome tanned leather based on emulsion nano-particles of styrene/butyl acrylate copolymers. N. Y. Sci. J..

[45] Sivasubramanian S., Manohar B.M., Rajaram A., Puvanakrishnan R. (2008). Ecofriendly lime and sulfide free enzymatic dehairing of skins and hides using a bacterial alkaline protease. Chemosphere.

[46] Xu W., Zhou J., Wang Y., Shi B. (2016). Modification of leather split by in situ polymerization of acrylates. Int. J. Polym. Sci..

